# Supplementary material for: DMRforPairs: identifying Differentially Methylated Regions between unique samples using array based methylation profiles
Source: BMC Bioinformatics. 2014 May 15;15:141. doi: 10.1186/1471-2105-15-141 (PMC4046028; doi:10.1186/1471-2105-15-141)
Supplement: Additional file 2 — DMRforPairs output for the comparison of A431-MCF7 and NA17018-NA17105. Please start from the HTML files in each folder. Available via the BMC Bioinformatics website. [file 1471-2105-15-141-S2.zip › 1394847754114233_MOESM2_ESM/A431_MCF7/figures/34699.pdf]

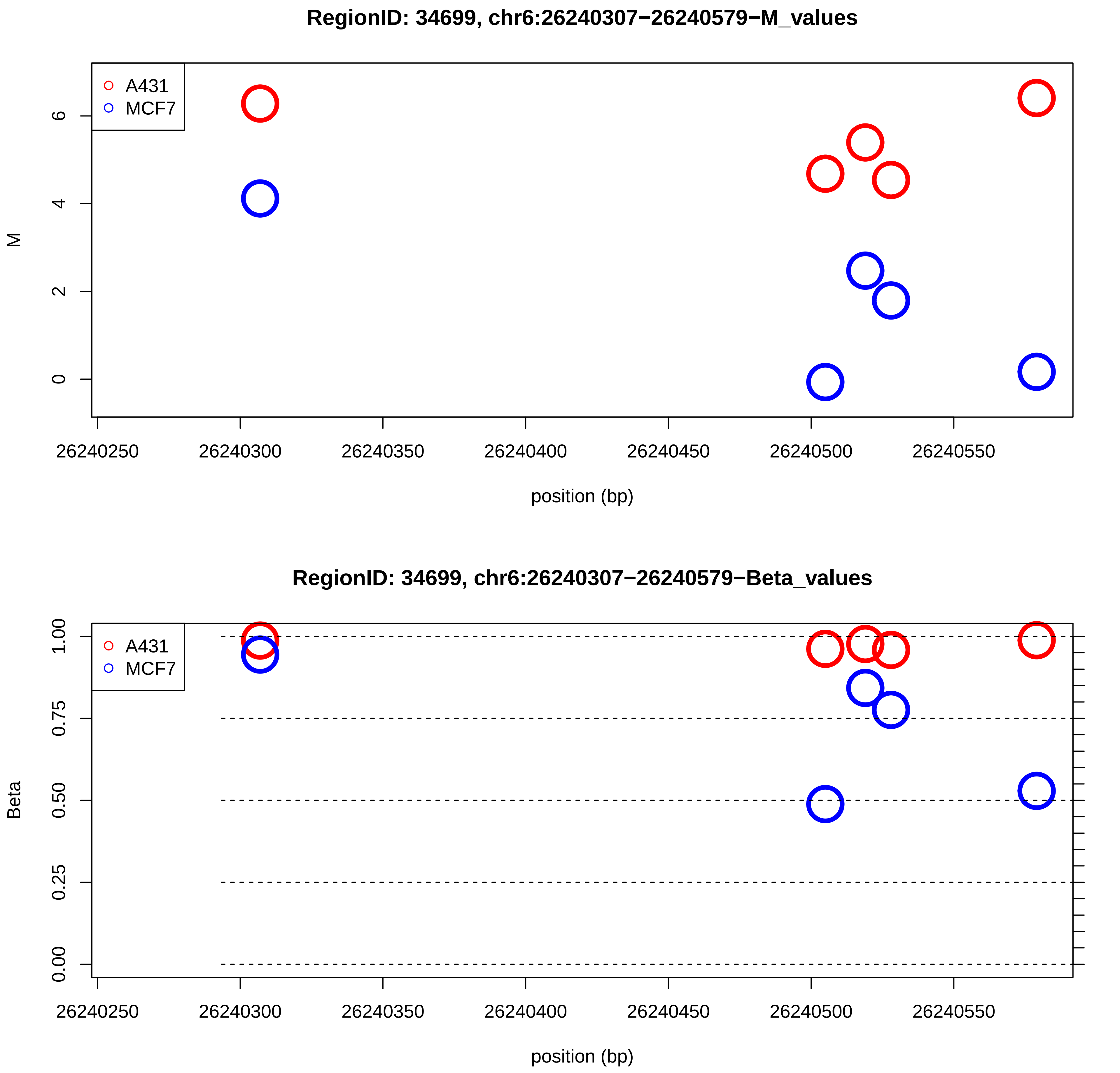
RegionID: 34699, chr6:26240307−26240579−M_values
A431
MCF7
M
0
2
4
6
26240250
26240300
26240350
26240400
26240450
26240500
26240550
position (bp)
RegionID: 34699, chr6:26240307−26240579−Beta_values
A431
MCF7
Beta
0.00
0.25
0.50
0.75
1.00
26240250
26240300
26240350
26240400
26240450
26240500
26240550
position (bp)

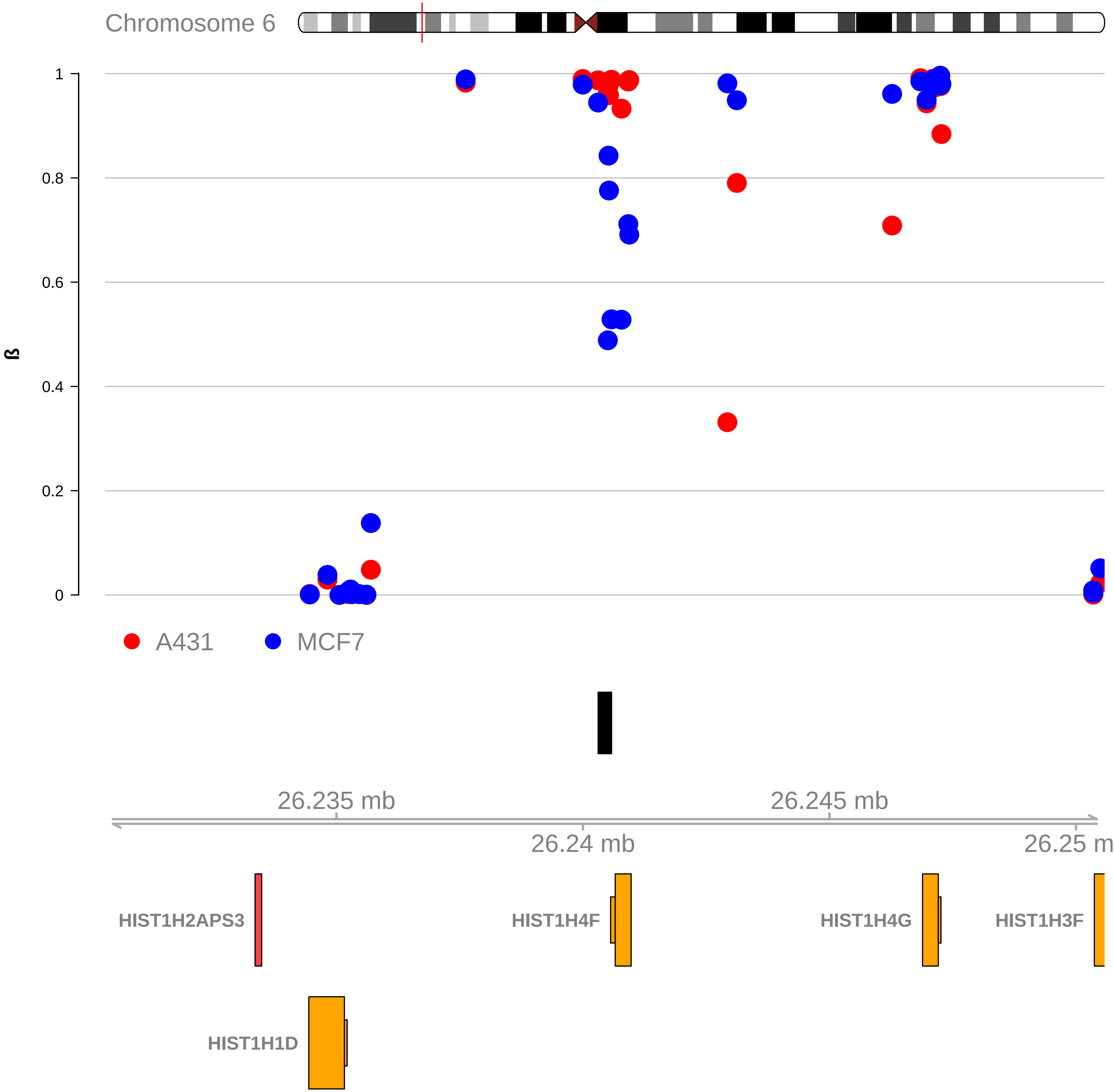

Chromosome 6
1
0.8
0.6
0.4
0.2
0
ß
A431
MCF7
26.235 mb
26.245 mb
26.24 mb
26.25 m
HIST1H2APS3
HIST1H4F
HIST1H4G
HIST1H3F
HIST1H1D
